# Rehabilitation in Pediatric Patients with Acute and Subacute Acquired Brain Injury: A Scoping Review on Non-Pharmacological Interventions

**DOI:** 10.3390/jcm15197645

**Published:** 2026-10-02

**Authors:** Davide Cardile, Giulia Marafioti, Giuliana Tanner, Irene Cappadona, Maria Pagano, Francesco Corallo, Carmela Rifici, Angelo Quartarone, Rocco Salvatore Calabrò

**Affiliations:** 1 IRCCS Centro Neurolesi Bonino Pulejo, S.S. 113 Via Palermo, C. da Casazza, 98124 Messina, Italy; 2 Dipartimento di Psicologia Generale, Università degli Studi di Padova, Via Venezia 8, 35131 Padova, Italy

**Keywords:** pediatric, adolescent, acquired brain injury, neurorehabilitation, non-pharmacological rehabilitation, disorder of consciousness

## Abstract

**Background:** Acquired brain injury in children and adolescents may lead to long-term neurological, cognitive, motor, and behavioral impairments. However, pediatric-specific evidence on rehabilitation strategies remains limited. **Objective:** This review aimed to summarize current evidence on non-pharmacological neurorehabilitation interventions for children and adolescents with acute/subacute acquired or traumatic brain injury, including a subset of patients with disorders of consciousness. **Methods:** A literature search was conducted in PubMed, Embase, Web of Science, and Scopus. Eligible studies investigated non-pharmacological rehabilitation approaches in pediatric or adolescent patients with severe acquired brain injury, traumatic brain injury, stroke, or disorders of consciousness. Data on interventions, outcomes, and main findings were extracted. **Results:** The reviewed studies included heterogeneous interventions, such as multidisciplinary rehabilitation, constraint-induced movement therapy, virtual-reality-based training, speech therapy, parent–child interaction therapy, sensory stimulation, autonomic monitoring, and neuromodulation. Overall, these approaches were feasible and generally well tolerated, with reported improvements in motor, functional, behavioral, autonomic, and consciousness-related outcomes. **Conclusions:** Non-pharmacological rehabilitation may support recovery in children and adolescents with severe brain injury, although current evidence remains preliminary and heterogeneous. Further multicenter studies with standardized protocols and outcome measures are needed to guide pediatric-specific rehabilitation practice.

## 1. Introduction

Acquired brain injury (ABI), encompassing both traumatic and non-traumatic etiologies, remains one of the leading causes of death and long-term disability among children and adolescents worldwide [1]. Recent meta-analytic data estimate a global incidence of pediatric traumatic brain injury (TBI) of approximately 226 cases per 100,000 children annually, with historical country-level estimates ranging from 47 to 280 per 100,000 depending on region, age group, and case-ascertainment methodology [2]. Broader epidemiological surveys suggest that more than three million children are affected by TBI each year globally, with incidence rates spanning from roughly 12 per 100,000 in some Nordic cohorts to nearly 280–486 per 100,000 in other national registries, and with low- and middle-income countries bearing a disproportionate share of both incidence and case fatality due to limited trauma infrastructure and underreporting [3,4]. Advances in acute neurocritical care have improved survival after severe pediatric brain injury, yet a substantial proportion of survivors are left with long-lasting cognitive, motor, and behavioral sequelae that interrupt normal developmental trajectories and impose a considerable cumulative burden on children, families, and healthcare systems over decades of life [5,6].

Despite this burden, the pharmacological management of pediatric ABI remains poorly supported by evidence. Age-specific randomized trials are scarce, and most pharmacological recommendations for children are extrapolated from adult protocols, an approach increasingly recognized as inadequate given the marked age-dependent differences in pharmacokinetics and pharmacodynamics [7] that characterize the developing central nervous system. The immature brain undergoes ongoing synaptogenesis, receptor expression changes, and neurotransmitter system maturation that extend well into the second decade of life, meaning that a drug’s action, tolerability, and even its long-term neurodevelopmental consequences can differ substantially from what is observed in adults, and can vary further depending on the child’s age and developmental stage at the time of exposure [8]. Amantadine, the pharmacological agent with the strongest evidence base for accelerating functional recovery in adult traumatic DoC, illustrates this problem well: pediatric case series report adverse events (including hallucinations, delusions, agitation, and mood disturbances) in roughly 9–16% of treated children, prompting clinicians to use it cautiously and often only when non-pharmacological alternatives have been exhausted [9]. More broadly, systematic reviews of pharmacological treatment for pediatric TBI and post-concussive symptoms conclude that the available evidence is scarce, heterogeneous, and insufficient to support any specific pharmacological standard of care across the pediatric age range [10]. Taken together, the combination of unpredictable neuropharmacological responses in a still-developing brain, a persistently limited efficacy signal, and safety concerns particular to younger patients justifies prioritizing non-pharmacological rehabilitative strategies as the mainstay of pediatric neurorehabilitation [11].

Within the non-pharmacological domain, cognitive, behavioral, and metacognitive rehabilitation constitutes a distinct and extensively studied body of literature. Laatsch et al. [12,13] published two comprehensive evidence-based reviews on this topic, encompassing a substantial number of pediatric studies (1980–2006 and 2006–2017, respectively) and proposing formal practice guidelines and recommendations for attention, memory, executive functioning, emotional/behavioral regulation, and family-centered treatment. Given the breadth and rigor of this existing synthesis, readers are referred to these works for a detailed appraisal of cognitive and behavioral rehabilitation in pediatric ABI. Beyond the aforementioned rehabilitative interventions, the other non-pharmacological approaches applied in pediatric ABI and disorders of consciousness (DoC) are themselves remarkably heterogeneous, spanning interventions with very different physiological mechanisms and putative modes of action. At one end of the spectrum, structured sensory and verbal stimulation programs (including auditory-based training, multisensory stimulation, family-centered affective stimulation, and motor-based therapy) aim to increase environmental input in order to enhance arousal, attention, and experience-dependent plasticity, and are widely used because of their low risk and ease of bedside implementation [14]. At the other end, advanced neuromodulation techniques act through direct modulation of neural excitability or network connectivity: non-invasive approaches such as transcranial direct current stimulation (tDCS), repetitive transcranial magnetic stimulation (rTMS), transcutaneous auricular vagus nerve stimulation, median nerve stimulation, and low-intensity focused ultrasound, alongside invasive options such as deep brain stimulation and spinal cord stimulation, target distinct cortical, subcortical, or peripheral pathways implicated in the maintenance of conscious awareness [15,16]. Meta-analytic evidence in adults indicates that these techniques are not interchangeable in efficacy: rTMS and peripheral nerve stimulation approaches have shown medium effect sizes on standardized consciousness scales, whereas tDCS has generally failed to outperform sham stimulation, underscoring that mechanism-of-action differences translate into meaningfully different clinical outcomes [17,18]. This mechanistic and procedural diversity (ranging from passive sensory enrichment to active, targeted neuromodulation) means that studies of “non-pharmacological interventions” in pediatric ABI cannot be treated as a single, homogeneous category when synthesizing evidence of benefit.

A further, often underappreciated source of variability lies within the pediatric population itself. Neonates, young children, and adolescents differ substantially in neuroanatomical maturation, synaptic density, myelination status, and baseline capacity for structural and functional reorganization, and these differences shape both the pathophysiological response to injury and the potential for recovery [17,19].

Comparative evidence suggest that the relationship between age at injury and functional recovery is complex and non-linear [14]. Although the developing brain may retain substantial capacity for neural reorganization early brain injury can also disrupt ongoing maturation and lead to slower or poorer long-term functional outcomes, particularly after severe or diffuse insults [20]. Consequently, the same non-pharmacological intervention (whether sensory stimulation or a specific neuromodulation protocol) cannot be assumed to act uniformly across a population encompassing infants, school-age children, and adolescents; treatment responses are likely to vary considerably according to the child’s developmental stage at the time of both injury and intervention [21,22].

Taken together, these considerations highlight an urgent need to characterize how non-pharmacological rehabilitative strategies have been applied and evaluated in children and adolescents with acute/subacute ABI. Through this scoping review we aim to synthesize current knowledge on how non-pharmacological interventions have been applied for children and adolescent with acute/subacute ABI or TBI (focusing on motor, sensory/autonomic, consciousness-related, and neuromodulatory interventions), including a subset of patients with disorders of consciousness, and to evaluate their impact on patient outcomes. Our goal is to inform clinical practice by identifying which rehabilitative approaches show evidence of benefit in this vulnerable population, and to highlight areas where further research is needed to develop effective, evidence-based pediatric neurorehabilitation protocols.

## 2. Materials and Methods

A review of currently published studies was performed in the following databases: Pubmed, Embase, Web of Science, Scopus. The search was carried out using the following search string: ((((pediatric[Title/Abstract]) OR (adolescent[Title/Abstract])) AND (stroke[Title/Abstract])) OR (brain injury[Title/Abstract])) AND (neurorehabilitation[Title/Abstract]).

Initially all articles were reviewed based on titles and abstracts by two investigators (D.C., G.M.), who independently collected data to minimize the risk of bias (e.g., publication bias, delay bias, language bias). Full-text articles deemed suitable for the study were then read by these researchers, and in case of disagreements regarding inclusion or exclusion criteria, a final decision was made by a third researcher (R.S.C.). PRISMA-ScR Checklist for reporting scoping reviews is available in the Supplementary Materials (File S1) and PRISMA (Preferred Reporting Items for Systematic Reviews and Meta-Analyses) diagram was added to describe the sequence of steps (identification, screening, eligibility, and inclusion) for the collection and determination of qualified studies as shown in Table 1. Disagreements between reviewers were resolved by consensus.

### 2.1. Inclusion Criteria

A study was included if it described or investigated non-pharmacological neurorehabilitative treatments in children and adolescent patients. Only studies conducted on human populations and published in English that met the following criteria: (I) articles involving patients aged between 1 and 26 years, (II) studies describing patients with stroke or severe acquired brain injury (both vascular and traumatic), (III) papers describing approaches, methods, treatments, or stimulation/neurorehabilitation devices, (IV) articles published in English.

### 2.2. Exclusion Criteria

A study was excluded if there was a lack of data or information about patients with severe acquired brain injury, and if it was single case report, dissertation, commentary, letter, or editorial. Even studies who exclusively address cognitive, behavioral, and metacognitive rehabilitation without a motor, sensory, autonomic, or neuromodulatory complementary treatment were excluded. Systematic, integrative, or narrative reviews were also excluded, although their reference lists were checked and included if appropriate.

## 3. Results

**Figure 1:**
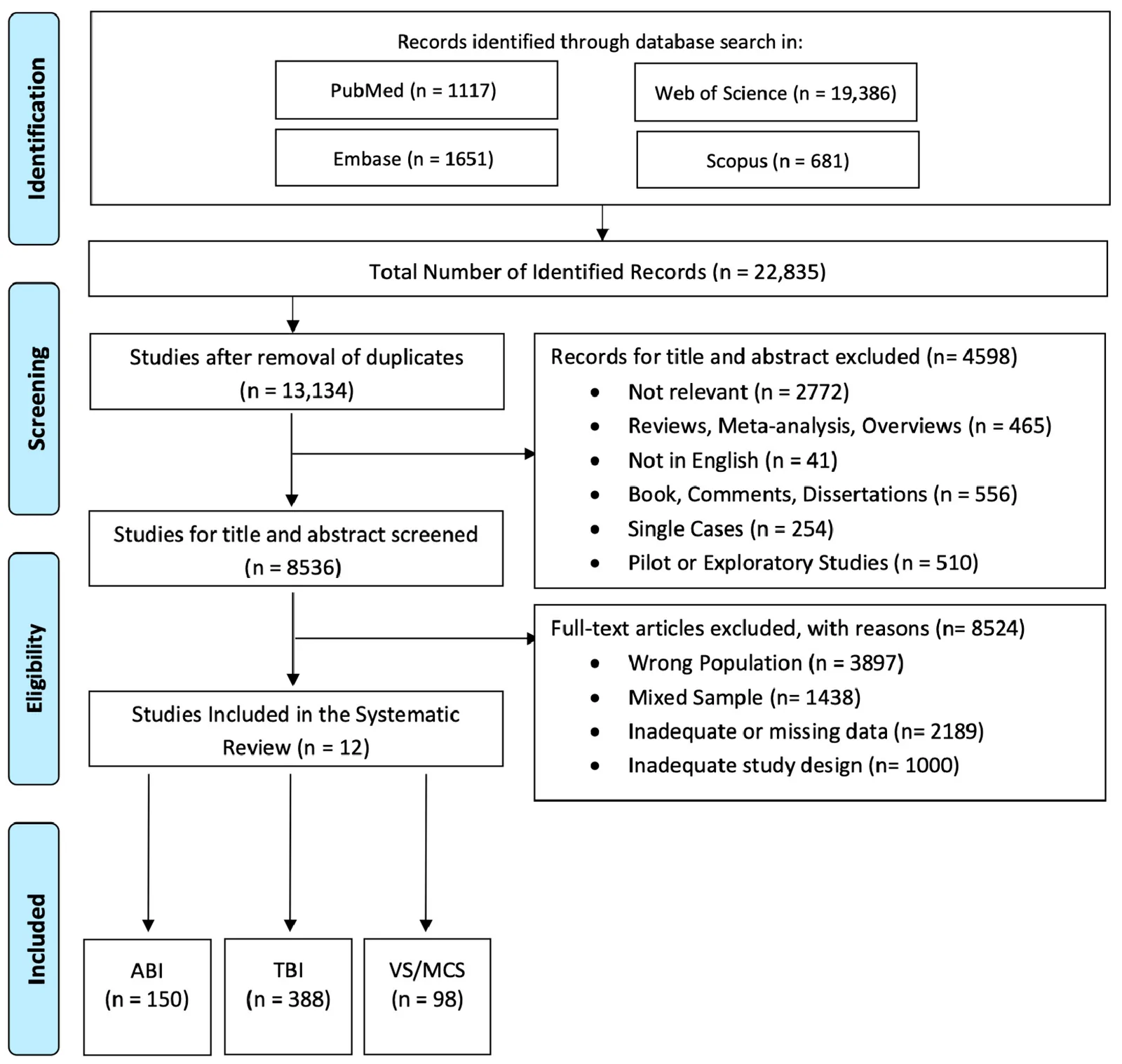
PRISMA Flow-chart for the current review.

A Data extraction was conducted on 22,835 articles. Of these, 13,134 were duplicates, and 41 were non-English language articles. The total number of full-text articles that were assessed for eligibility was 8536, and the number of studies included was finally 12 (Figure 1).

### 3.1. Study Characteristics

The final set of included studies comprised heterogeneous designs, ranging from small case series and observational cohorts to randomized controlled trials (Table 1).

Sample sizes varied widely from as few as 3 participants in single-center case series [24,27] to 254 patients in the largest cohort [28], reflecting substantial variability in the scale of available evidence. Most studies enrolled children and adolescents with moderate-to-severe TBI or ABI, often during the acute or subacute rehabilitation phase [23,24,25,26,27,28,29,30,31,32] while one trial specifically focused on children with unresponsive wakefulness syndrome (UWS) or minimally conscious state (MCS) after TBI [33]. Across studies, outcome assessment relied on a broad range of standardized tools capturing level of consciousness, functional independence, motor performance, behavior, quality of life, and neurophysiological indices (e.g., CRS-R, GCS, GOS/GOS-E Peds, WeeFIM, GMFM, QUEST, Besta Scale, PEDI, HRV parameters).

#### 3.1.1. Rehabilitation Interventions

The interventions evaluated were diverse and can be grouped into several main categories (Table 2).

Some studies investigated the effect of comprehensive multidisciplinary inpatient rehabilitation programs of varying intensity, typically combining physical therapy, occupational therapy, and speech-language pathology within individualized treatment plans [25,26,29,32]. Other work focused on more specific motor interventions, such as constraint-induced movement therapy (CIMT) for upper-limb hemiparesis after pediatric TBI [26] or virtual-reality based balance training using the Nintendo Wii Fit in children undergoing acute ABI rehabilitation [27]. Additional studies examined non-motor approaches targeting different functional domains. One case series explored speech therapy with electropalatography-based visual biofeedback for dysarthria following severe TBI [24], while another trial assessed parent–child interaction therapy (PCIT) for sleep problems and externalizing behaviors in children with TBI [34]. In the domain of sensory and autonomic modulation, early multimodal sensory stimulation (multimodal early onset stimulation, MEOS) was applied to patients comatose for more than 48 h after TBI [32], and standard inpatient rehabilitation was used as a context to study changes in cardiac autonomic control via heart rate variability (HRV) measures [28]. Finally, neuromodulation techniques were investigated in two studies: transcranial direct current stimulation (tDCS) combined with gamified attention training after pediatric ABI [30] and high-frequency repetitive transcranial magnetic stimulation (rTMS) over the left dorsolateral prefrontal cortex in children with DoC after TBI, both delivered alongside conventional rehabilitation [31].

#### 3.1.2. Clinical and Functional Outcomes

Overall, most studies reported improvements over time in at least some of the clinical or functional outcomes considered, although effect sizes and domains of change varied across interventions. In the motor domain, CIMT combined with physiotherapy was associated with significant gains in gross motor function, upper-limb performance (QUEST subscales), and Besta neurological scores, as well as favorable changes in 3D kinematic parameters, with 70% of children improving by at least one level on the Manual Ability Classification System (MACS) [27]. Virtual-reality balance training with Wii Fit was well tolerated, led to enhanced dynamic balance, and was accompanied by increased motivation and improvements in self-care and mobility, relative to baseline conventional therapy [24]. Within multidisciplinary inpatient programs, functional status commonly improved from admission to discharge. In one study, functional scales capturing daily activities and social-behavioral functioning showed significant gains with large effect sizes, and children classified as “most improved” exhibited greater change than those in the “least improved” group [26]. Another cohort comparing low- versus high-intensity multidisciplinary neurorehabilitation reported increased functional independence in both groups, with no significant difference between intensities [29]. A randomized trial of intensive versus conventional rehabilitation after moderate-to-severe TBI found that higher intensity accelerated early attainment of maximal Functional Independence Measure (FIM) and Glasgow Outcome Scale (GOS) scores, although this advantage did not translate into superior 12-month outcomes [32]. In the largest pediatric severe TBI cohort, inpatient rehabilitation after acute care was associated with more favorable global functional outcome in children with lower GCS at discharge, while quality-of-life scores did not significantly differ between rehabilitation groups [25]. Targeted interventions addressing speech and behavior also yielded domain-specific benefits. Electropalatography-based speech therapy improved consonant accuracy, phoneme duration, and intelligibility at word and phrase level, though generalization to everyday speech remained limited [23]. PCIT led to a reduction in sleep problems and externalizing behaviors in children with TBI, and these gains were maintained at two-month follow-up. In studies focusing on autonomic and sensory aspects, MEOS produced significant autonomic responses even in deep coma, with tactile and acoustic stimulation emerging as particularly effective components; at two-year follow-up, patients’ outcomes ranged from VS to good recovery (GOS 5), including some individuals who returned to work [33]. In a subacute TBI cohort, standard inpatient rehabilitation did not change resting HRV indices over eight weeks, but led to significant improvements in HRV responses to paced breathing and handgrip, along with better functional status [29].

#### 3.1.3. Neuromodulation and Safety

Evidence from neuromodulation studies was mixed. The within-subject, sham-controlled tDCS study found no overall advantage of active stimulation over sham on attention measures, although individual response appeared to depend on baseline functional connectivity and electric field strength; importantly, the intervention was well tolerated [30]. In contrast, the randomized controlled trial of 5 Hz rTMS over the left dorsolateral prefrontal cortex in children with DoC post-TBI showed greater improvements in Coma Recovery Scale-Revised (CRS-R), GCS, serum neuron-specific enolase (NSE), and consciousness level (transition from UWS/MCS to higher states) in the rTMS plus rehabilitation group compared with conventional rehabilitation alone, without reported adverse events [33].

## 4. Discussion

This scoping review synthesized the available evidence on non-pharmacological neurorehabilitation interventions for children and adolescents with severe brain injury and DoC or severe functional sequelae. Overall, the included studies indicate that a range of multidisciplinary and targeted rehabilitation approaches are feasible in this population and are generally associated with improvements in motor, functional, behavioral, and autonomic outcomes, although the quality of evidence remains low and heterogeneous [23,24,25,26,27,28,29,30,34]. Beyond documenting whether these approaches are associated with improvement, we tried to consider why and through which biological pathways non-pharmacological interventions may promote clinical recovery.

Across studies, multidisciplinary inpatient rehabilitation (often combining physical, occupational, and speech-language therapy) was associated with significant gains in functional independence and daily activities from admission to discharge. These findings are consistent with Bedell et al. [23], who showed that functional activity scales for pediatric brain injury inpatient rehabilitation are responsive to change and capture meaningful improvements over the course of a typical admission. Similarly, Zhu et al. [25] demonstrated in a randomized controlled trial including adolescents and adults with moderate-to-severe TBI that higher-intensity rehabilitation (4 h/day vs. 2 h/day) accelerated the attainment of maximal FIM and GOS scores in the first months post-injury, even though 12-month outcomes did not differ between groups. Evidence from pediatric cohort studies also supports the clinical relevance of inpatient rehabilitation and multidisciplinary treatment, although effects may vary according to baseline severity and therapy intensity [25,29]. Taken together, these data suggest that early, structured, and sufficiently intensive rehabilitation can speed functional recovery, even if long-term outcomes may ultimately be constrained by injury severity and other prognostic factors.

Targeted motor interventions such as constraint-induced movement therapy (CIMT) and virtual-reality-based balance training also showed promising results in pediatric severe brain injury. The CIMT program evaluated by Cimolin et al. [26] in children with TBI-related hemiplegia led to significant improvements in upper-limb function on GMFM, Besta Scale, QUEST, and MACS, along with favorable changes in 3D kinematic parameters, supporting the feasibility and efficacy of stringent CIMT protocols in pediatric ABI. This aligns with earlier work on CIMT in children with acquired brain injury, which demonstrated large effect sizes in upper-limb use and activities of daily living when child-friendly shaping exercises were integrated into intensive protocols. Likewise, Wii-based “Wii-habilitation” during acute inpatient rehabilitation improved dynamic balance and functional abilities in children with acquired brain injury and was reported to be safe and highly motivating [24], a key consideration in pediatric rehabilitation. These findings mirror broader evidence that low-cost virtual reality and gaming platforms can be effective adjuncts for balance and mobility recovery in acquired brain injury populations.

### 4.1. Speech, Behavior, and Autonomic Outcomes

Beyond gross motor function, the review identified interventions addressing speech, behavior, and autonomic regulation. Morgan et al. [24] showed that electropalatography-based biofeedback can improve articulatory precision and intelligibility at word and sentence level in adolescents with dysarthria after severe TBI, although generalization to everyday speech remained limited. This is in line with the broader EPG literature, which supports its utility for targeting articulatory placement but highlights challenges in achieving durable functional gains in connected speech without extended or booster interventions. Parent–Child Interaction Therapy (PCIT), adapted to children with TBI and elevated externalizing behaviors, was associated with reductions in behavior problems and sleep difficulties that were maintained at follow-up, echoing feasibility data from intensive PCIT formats for early childhood TBI.

In the autonomic domain, multimodal early onset stimulation (MEOS) applied to patients in coma after severe brain injury produced measurable changes in vegetative parameters, particularly heart and respiratory rates, with tactile and acoustic stimulation emerging as the most effective components [32]. These findings are consistent with broader sensory stimulation literature, which suggests that multimodal stimulation can modulate arousal and autonomic responses in coma and VS, even though high-quality randomized evidence remains limited. Furthermore, subacute inpatient rehabilitation was associated with improved heart-rate-variability responses to paced breathing and handgrip in children with severe TBI [29], paralleling adult data showing depressed HRV after TBI with gradual normalization over the first months post-injury as autonomic function recovers.

### 4.2. Neuromodulation and Disorders of Consciousness

The included neuromodulation studies illustrate both the potential and limitations of non-invasive brain stimulation in pediatric acquired brain injury and DoC. In the reviewed trial, a short course of tDCS combined with gamified attention training did not yield a clear group-level advantage over sham stimulation on attention measures, but response appeared to vary with baseline functional connectivity and electric field strength, and the intervention was well tolerated [30]. This pattern reflects recent meta-analytic evidence in adult DoC indicating that tDCS can produce modest improvements in CRS-R scores, particularly in patients with traumatic etiology and minimally conscious state, while effects in VS/UWS are less robust.

By contrast, repetitive transcranial magnetic stimulation (rTMS) over the left dorsolateral prefrontal cortex, delivered in combination with conventional rehabilitation, was associated with greater gains in CRS-R, GCS, serum NSE, and transitions in consciousness level compared with rehabilitation alone in children with DoC after TBI [33]. These pediatric findings are broadly concordant with adult randomized, sham-controlled rTMS trials in DoC, which have reported improvements in CRS-R scores and higher rates of emergence from MCS when stimulation targets prefrontal or motor networks. However, adult response rates to rTMS in DoC remain around one-third of treated patients, and optimal protocols (frequency, intensity, target, and timing post-injury) are still under investigation. Thus, while neuromodulation represents a promising avenue to enhance recovery in selected pediatric patients, current evidence supports its use only in specialized centers and in the context of rigorous outcome monitoring.

### 4.3. Clinical Findings, Underlying Mechanisms and Future Directions

A plausible biological explanation for the improvements reported by the studies included in this review is experience-dependent neuroplasticity, namely the capacity of the nervous system to modify synaptic efficacy, structural connectivity, and functional organization in response to injury and repeated experience [35,36]. This process may be particularly relevant in pediatric populations because brain maturation, synaptogenesis, and myelination are still ongoing; however, developmental plasticity should not be equated automatically with a uniformly favorable prognosis, since early injury may also disrupt subsequent maturation [37,38]. In this context, repeated and meaningful practice (such as CIMT, virtual-reality training, intensive multidisciplinary therapy, speech biofeedback, and family-mediated behavioral intervention) may reinforce residual networks and promote the recruitment of spared cortical and subcortical resources. The clinical rationale is therefore not simply to increase the amount of stimulation, but to deliver appropriately dosed, salient, and goal-directed experiences capable of engaging the neural systems relevant to the impaired function.

At the systems level, recovery may involve cortical remapping, or functional reorganization, through which spared peri-lesional, contralesional, or distributed networks assume part of the function of damaged regions [35]. Such reorganization is thought to be driven by repetition, behavioral relevance, feedback, and reward, all central elements of CIMT and game-based rehabilitation [24,27]. At the synaptic level, activity-dependent plasticity may include the unmasking of subthreshold connections and the recruitment of so-called silent synapses. Silent synapses are structurally present synaptic contacts that are functionally inactive at resting membrane potentials, classically because they express NMDA but not functional AMPA receptors [39]. Experimental and translational literature suggests that injury and repeated neural activity can modify the availability and efficacy of these connections, thereby providing a potential substrate for the recovery of impaired motor and cognitive functions [40]. Although direct evidence of silent-synapse reactivation in pediatric DoC is not available, this mechanism provides a biologically plausible explanation for why repeated sensory, motor, and neuromodulatory input may progressively strengthen residual circuits rather than merely produce transient behavioral effects. These mechanisms are particularly relevant to patients in VS, now commonly termed UWS, in whom recovery requires not only restoration of specific motor or cognitive functions but also sufficient recovery of arousal and large-scale network integration. The ARAS, which links brainstem arousal nuclei with the thalamus, basal forebrain, and cerebral cortex, is fundamental to maintaining wakefulness and enabling conscious processing [20,41,42]. The mesocircuit hypothesis proposed by Schiff [43] further posits that severe brain injury can cause functional downregulation of an anterior forebrain network involving the central thalamus, striatum, globus pallidus, and frontal cortex; re-establishing excitatory drive within this circuit could facilitate recovery of sustained cortical activation and observable responsiveness. In support of this model, Schiff et al. [44] showed that central thalamic deep brain stimulation was associated with improved behavioral responsiveness in a patient who had remained in a minimally conscious state for six years, while Laureys et al. [45] reported restoration of thalamocortical connectivity following recovery from persistent VS.

Within this framework, the effects of sensory stimulation and neuromodulation can be interpreted as partly dependent on the integrity of residual arousal and thalamocortical pathways. In the MEOS study, tactile and acoustic stimulation elicited measurable autonomic responses in deeply comatose patients and were considered the most consistently active components of the protocol [32]. Such responses cannot be considered proof of awareness or a favorable prognosis; nevertheless, they indicate preservation of physiological reactivity and support the rationale for carefully individualized, non-overstimulating sensory programs. Repeated tactile, acoustic, vestibular, and proprioceptive inputs may increase afferent drive to residual brainstem–thalamic–cortical networks and thereby support arousal regulation, especially when stimulation is adjusted to the patient’s behavioral and autonomic tolerance [32]. In parallel, the improvement in heart-rate-variability responses to autonomic challenges after subacute rehabilitation in children with severe TBI suggests partial restoration of autonomic adaptability, although resting autonomic indices remained impaired [29].

The neuromodulation findings further illustrate the importance of network integrity and intervention specificity. The pediatric rTMS trial targeting the left dorsolateral prefrontal cortex reported larger improvements in CRS-R, GCS, serum NSE, and level of consciousness when rTMS was combined with conventional rehabilitation than with rehabilitation alone [33]. This result is consistent with the hypothesis that high-frequency stimulation may increase cortical excitability and facilitate communication within fronto-parietal and thalamocortical networks implicated in conscious processing [46]. Conversely, the sham-controlled pediatric tDCS study did not demonstrate a group-level advantage on attentional outcomes, although response variability was related to baseline connectivity and modeled electric-field strength [30]. This finding is compatible with evidence from adult DoC suggesting that tDCS effects are modest and variable, with less consistent benefit in VS/UWS than in minimally conscious state [47]. Accordingly, the available data do not support considering all neuromodulatory approaches as equivalent: the clinical effect is likely to depend on the target, stimulation parameters, timing, lesion characteristics, and, above all, preservation of the networks that the intervention intends to modulate. The views advanced by the cited authors can therefore be integrated into a single multilevel account of recovery. Authors of conventional and motor-rehabilitation studies mainly emphasize the behavioral consequences of structured, repetitive, task-specific experience and its capacity to drive cortical reorganization [24,26,27]. Basic and translational neuroscience studies identify synaptic strengthening, unmasking of latent connections, and possible recruitment of silent synapses as cellular mechanisms that can stabilize these gains [39,40]. In the specific setting of DoC, Schiff and colleagues [43,44], as well as Edlow et al. [42], emphasize recovery of the ARAS, central thalamus, and thalamocortical mesocircuit as a necessary network-level condition for sustained arousal and the re-emergence of conscious behavior. Thus, non-pharmacological rehabilitation may operate along a continuum: sensory enrichment and conventional therapy may capitalize on residual spontaneous plasticity, while targeted neuromodulation may more directly engage cortical and thalamocortical nodes of the arousal network.

A formal quantitative comparison or ranking of the included interventions is not appropriate because of the marked heterogeneity in diagnoses (TBI, ABI, stroke, and DoC), clinical severity, age and developmental stage (from infants and young children to adolescents) treatment protocols, dosage, timing after injury, and outcome measures. Nevertheless, a cautious qualitative appraisal is possible. Multidisciplinary inpatient rehabilitation and task-specific motor interventions, particularly CIMT, provided the most consistent evidence of functional benefit across the broader pediatric ABI population [16,18,19,21]. MEOS and rTMS showed the most direct signal for consciousness-related outcomes in patients with DoC, although the evidence base remains small and the specific contribution of the intervention cannot be fully separated from concurrent conventional rehabilitation [32,33]. tDCS showed no clear group-level clinical benefit in the included pediatric study [30,47], while EPG-based speech therapy and PCIT appeared useful for circumscribed targets (speech intelligibility and behavioral/sleep outcomes, respectively) rather than for global recovery [24,26]. This qualitative assessment should be considered hypothesis-generating and should not be interpreted as proof of comparative superiority. Several limitations must be considered. This review focused on motor, sensory/autonomic, consciousness-related, and neuromodulatory interventions in the acute/subacute phase, and did not systematically capture the parallel literature on cognitive, behavioral, and family-centered rehabilitation. Moreover, most included studies were single-center reports, small case series, or non-concurrent observational designs, and only a minority used randomized controlled methods or blinded outcome assessment. The timing, intensity, content, and duration of intervention varied substantially, as did the clinical outcomes, which ranged from global functional measures to kinematic, neurophysiological, and autonomic indices. In addition, many studies did not stratify findings according to etiology, DoC diagnosis, or age category, despite the likely relevance of these variables to prognosis and treatment responsiveness. These limitations, together with the inability to separate treatment effects from natural recovery in several designs, prevent firm causal inferences and limit direct cross-study comparison.

## 5. Conclusions

Current evidence suggests that non-pharmacological rehabilitation may support functional recovery in children and adolescents with severe brain injury, although the literature remains preliminary and heterogeneous in terms of study designs, protocols, and outcome measures. Pediatric rehabilitation programs should prioritize early, individualized, developmentally appropriate, and task-oriented interventions, while retaining the flexibility to address motor, cognitive, communicative, behavioral, and autonomic needs. Family involvement and the home-based components of CIMT may enhance treatment feasibility and promote generalization to everyday contexts. For patients with low levels of consciousness, structured sensory stimulation and autonomic monitoring can be integrated into specialized multidisciplinary care, with careful interpretation of physiological responses and avoidance of overstimulation. Neuromodulation, particularly rTMS, represents a promising adjunctive strategy but should currently be implemented only in specialized settings with rigorous safety procedures and longitudinal clinical and neurophysiological monitoring. Future multicenter studies employing standardized protocols and repeated pediatric outcome measures and stratifying samples by age, diagnosis, injury mechanism, time post-injury, and biomarkers of residual network integrity are needed to determine which interventions are effective, for which patients, and at which phase of recovery, while clarifying the underlying neuroplastic, synaptic, and ARAS–thalamocortical mechanisms of clinical improvement.

## Figures and Tables

**Table 1:** Characteristics of patients enrolled by studies included in the review.

Study	Patients	Neurological Deficits	Follow-Up	Clinical Scales
Bedell et al., 2002 [23]	65 (ABI)	Deficits in mobility, personal care, communication and social-behavioral function	36 days	GCS, prototype CPM (daily activity + social-behavioral subscales); 7-point GCR Scale
Morgan et al., 2007 [24]	3 (TBI)	Persistent dysarthria	No	GCS, MAYO Dysarthria Rating Scale, GFTA-2, WASI, CSIM, ASSIDS, IRS-MSD
Zhu et al., 2007 [25]	68 (TBI)	Impairments in self-care, mobility, language, memory, calculation, and reasoning	1–6, 8, 10, 12 months	GCS, FIM, GOS, NCSE
Cimolin et al., 2012 [26]	10 (TBI)	Hemiplegia of the upper limb, mild-to-moderate cognitive impairment, visual disorders in 7/10 patients	No	GCS, GOS, QUEST, Besta Scale, GMFM, MACS
Tatla et al., 2014 [27]	3 (ABI)	Impaired balance, mobility and functional independence. Impaired cognition or memory	No	Ranchos, TUG, MFRT, COP (Wii Balance Board), PMS, PEDI
Gao et al., 2022 [28]	254 (TBI)	Global functional disability and impaired consciousness at hospital discharge	12 months	GCS, GOS-E Peds, PedsQL, FSS, AIS, ISS, PRISM III
Sorek et al., 2023 [29]	33 (TBI)	Impaired autonomic regulation, reduced grip strength, functional dependence and reduced global functional status.	8 weeks	GCS, GOS-E Peds, WeeFIM; HRV (SDNN, RMSSD, LF, HF, LF/HF ratio) via Polar RS800CX
Stein et al., 2025 [30]	14 (TBI) + 1 (ABI)	Impaired attention, inhibitory control and executive regulation, with elevated parent-reported BRIEF-2 Behaviour Regulation	No	NIH Flanker RT, SSRT (Stop Signal Task); CNS Vital Signs (NCI, RT, Simple/Complex Attention); BRIEF2 (BRI, Inhibit, Shift); AEQ
Furman et al., 2026 [31]	81 (ABI)	Impaired functional independence across self-care, mobility, communication, social cognition and other cognitive abilities.	n.a.	WeeFIM
Lippert-Grüner & Terhaag, 2000 [32]	89 (ABI)	Impaired verbal and motor responses and reduced consciousness.	2 years	WeeFIM, GOS-E Peds
Zheng et al., 2025 [33]	31 (UWS) + 67 (MCS)	Profound impairment of arousal, awareness, command following, communication and functional interaction	No	CRS-R, GCS, serum NSE; consciousness level classification (UWS/MCS/EMCS)
Smith et al., 2025 [34]	6 (TBI)	Elevated sleep problems and clinically significant disruptive, aggressive or attention-related behaviour.	2 months	CBCL, ECBI

Legend: acquired brain injury (ABI); Abbreviated Injury Scale (AIS); Assessment of Intelligibility of Dysarthric Speech (ASSIDS); Adverse Effects Questionnaire (AEQ); Behavior Rating Inventory of Executive Function, 2nd edition (BRIEF2) subscales: Behavior Regulation Index (BRI), Inhibit, Shift; Child Behavior Checklist (CBCL); CNS Vital Signs subscales: Neurocognitive Index (NCI), Reaction Time (RT); Coma Recovery Scale-Revised (CRS-R); center of pressure (COP); Children’s Speech Intelligibility Measure (CSIM); Clinical Performance Measure (CPM); Eyberg Child Behavior Inventory (ECBI); emergence from minimally conscious state (EMCS); Functional Independence Measure (FIM); Functional Status Scale (FSS); Glasgow Coma Scale (GCS); Glasgow Outcome Scale (GOS); Glasgow Outcome Scale—Extended for Pediatrics (GOS-E Peds); Global Change Rating Scale (GCR Scale); Goldman-Fristoe Test of Articulation-2 (GFTA-2); Gross Motor Function Measure (GMFM); heart rate variability (HRV) parameters: standard deviation of N-N intervals (SDNN), root mean square of successive differences (RMSSD), low frequency (LF), high frequency (HF); Injury Severity Score (ISS); Intelligibility Rating Scale for Motor Speech Disorders (IRS-MSD); Manual Ability Classification System (MACS); minimally conscious state (MCS); Modified Functional Reach Test (MFRT); neuron-specific enolase (NSE); Neurobehavioral Cognitive Status Examination (NCSE); Pediatric Evaluation of Disability Inventory (PEDI); Pediatric Motivation Scale (PMS); Pediatric Quality of Life Inventory (PedsQL); Pediatric Risk of Mortality III (PRISM III); Pediatric Rancho Los Amigos Level of Consciousness Scale (Ranchos); Quality of Upper Extremities Skills Test (QUEST); stop signal reaction time (SSRT); traumatic brain injury (TBI); Timed Up and Go test (TUG); unresponsive wakefulness syndrome (UWS); Wechsler Abbreviated Scale of Intelligence (WASI); Functional Independence Measure for Children (WeeFIM); Not assessed (n.a.).

**Table 2:** Summary of aim, treatment characteristics and results of studies included.

Study	Aim	Treatment	Temporal Features	Treatment Paradigm	Main Results
Bedell et al., 2002 [23]	To describe conceptual/methodological issues in developing a responsive clinical performance measure for pediatric brain-injury inpatient rehabilitation, illustrated through responsiveness analyses of two prototype activity scales	Standard multidisciplinary inpatient rehabilitation (OT, PT, speech-language pathology)	Retrospective chart analysis from admission to discharge; mean length of stay 36 days (range 5–217 days) across 8 US sites (data collected 1998–1999)	Physician, nurse, OT, PT, speech pathologist, psychologist, social worker) of admission/discharge charts using a 19-item Daily Activity scale and 10-item Social-Behavioral Activity scale, scored via Rasch partial-credit rating-scale model (0–100 scale); an external 7-point global-change rating was used as a validity criterion	The Daily Activity score increased from 22.20 (SD 18.68) to 46.26 (SD 19.27), with a large effect size of 1.29 (*p* < 0.001). The Social-Behavioral score rose from 33.59 (SD 24.09) to 54.18 (SD 20.03), with an effect size of 0.85 (*p* < 0.001). All individual items improved significantly (*p* < 0.001, Wilcoxon). Participants in the most-improved versus least-improved groups differed significantly, with effect sizes of 1.12 for Daily Activity and 1.58 for Social-Behavioral (*p* < 0.001). Improvements showed moderate correlations with external global-change ratings (r = 0.47 for Daily Activity and r = 0.58 for Social-Behavioral; *p* < 0.01).
Morgan et al., 2007 [24]	To determine whether electropalatography (EPG)-based treatment improves perceptual speech intelligibility, articulatory accuracy and tongue-to-palate coordination in children with persistent post-TBI dysarthria.	Electropalatography-based articulation therapy.	Duration: 12 weeks. Frequency: 10 sessions, approximately once weekly. Intensity: 45 min per session, delivered by an experienced speech-language pathologist. No home-based EPG programme was provided.	Each participant wore a custom acrylic palatal mould with embedded electrodes. Tongue-to-palate contacts were displayed in real time using the Reading WinEPG/EPG-3 system, with simultaneous acoustic recording. Treatment targeted /t/, /k/, and /s/ across six hierarchical phases: familiarization/visual self-monitoring; silent tongue-to-palate posturing; manner/voicing contrasts; isolated phonemes/syllable sequences; CVC words/phrases; and carry-over into conversation. Progression required 10/10 acoustically and spatially accurate productions at each level. Training emphasized accurate lingual placement and reduced articulatory overshoot, not direct timing drills.	Perceptual dysarthria improved in all cases: consonant imprecision and prolonged phoneme duration decreased (milder ratings), and vowel distortion improved in Case 3. Single-word intelligibility was unchanged in Case 1 (96%) but increased in Cases 2 (from 82%to 94%) and 3 (from 76% to 92%); sentence intelligibility improved in all (Case 1: from 84% to 92%; Case 2: from 80% to 90%; Case 3: from 74% to 84%). Parent-rated functional intelligibility improved by one level in Cases 2 and 3. EPG showed more accurate/stable tongue placement, reduced excessive contact/double articulation, better /k/ sealing, less lateral escape for /s/, and lower phoneme variability. No consistent reduction in total phoneme duration emerged, suggesting timing was not reliably modified by this spatially focused treatment.
Zhu et al., 2007 [25]	To determine, via a prospective assessor-blind RCT, whether increasing the intensity of early post-traumatic rehabilitation improves the functional outcome of patients with moderate-to-severe TBI	Intensive multidisciplinary rehabilitation program (PT, OT, speech therapy) vs. conventional-intensity rehabilitation (same content, different dosage)	Duration: up to 6 months (max), assessments monthly to month 6 then bimonthly to month 12. Frequency: 5 days/week for both groups. Intensity: intensive group 4 h/day vs. control group 2 h/day	Both groups received identical multidisciplinary training in quality and content (physical/sensori-motor training, functional/ADL re-training, psycho-social/social-skills training, and speech therapy) quantified in 1-h units. The control group received 2 units in the morning (1 PT + 1 OT) plus 1 unit of speech therapy. The intensive group received an additional 2 units in the afternoon (1 PT + 1 OT) plus 2 units of speech therapy. Case therapists upgraded programs using identical, blinded criteria, and discharge decisions were made by a neurosurgeon blinded to group allocation.	The primary outcome, total FIM score, improved from 46 (SD 28) to 115 (SD 27) at 6 months and 116 (SD 27) at 12 months in the intensive group, and from 52 (SD 32) to 117 (SD 16) and 119 (SD 11) in the control group; between-group differences were non-significant (repeated-measures ANOVA, *p* = 0.775). However, a significantly higher proportion in the intensive group achieved the maximum FIM score at month 3 (47% vs. 19%, *p* = 0.016). The intensive group also showed significantly higher rates of maximum GOS (good recovery) at months 2 (28% vs. 8%, *p* = 0.034) and 3 (38% vs. 14%, *p* = 0.044), although these differences diminished after month 6. No significant between-group differences emerged for NCSE cognitive scores or length of hospital stay.
Cimolin et al., 2012 [26]	To quantify upper-limb movement impairment in hemiplegic children with TBI using clinical scales and 3D kinematics, and to evaluate quantitatively the effectiveness of a CIMT program on upper-limb function	Modified Constraint-Induced Movement Therapy (CIMT)	Duration: 10 consecutive weeks. Frequency: 7 days/week (glove worn daily); structured clinic sessions 3/week. Intensity: 3 h/day (1.5 h therapist-led + 1.5 h parent-led at clinic sessions; full 3 h parent-led at home on the other 4 days)	Restraining fabric glove with rigid volar splint immobilizing the unaffected hand/thumb; unimanual training targeting 4 domains: perceptual-motor activities, reach-grasp-hold-manipulate tasks, postural/balance activities, and self-care/ADL tasks (e.g., puzzles, cards, using utensils, dusting); clinic sessions supervised by a therapist, home sessions supervised by trained parents.	GMFM improved from 88.1 to 92.6 (*p* < 0.05). QUEST total score increased from 65.8 to 71.1 (*p* < 0.05), with gains in both dissociated movement (from 63.5 to 69.3) and grasp (from 59.2 to 67.5). Besta grip (from 6.9 to 8.7) and bilateral manipulation (from 4.3 to 5.8) also improved (all *p* < 0.05, medium–large effect sizes). Kinematic analysis of the affected limb showed reduced total movement duration (from 3.8 to 2.5 s), increased mean velocity (from 0.3 to 0.5 m/s), and decreased adjusting sway (from 253.7 to 82.9 mm; all *p* < 0.05). Seventy percent of children improved by one MACS level.
Tatla et al., 2014 [27]	To evaluate whether daily Nintendo Wii-Fit balance training improves static and dynamic balance, motivation to participate in rehabilitation and functional ability in children undergoing acute rehabilitation after acquired brain injury, compared with traditional balance therapy.	Nintendo Wii-Fit balance therapy compared with individualized traditional balance rehabilitation.	Study duration: 4 weeks. Frequency: 5 consecutive days per week. Intensity: 30 min of balance training per day. Intervention duration varied by randomized protocol: 8, 12 or 15 days of Wii training. Traditional balance therapy was delivered for the same daily duration during baseline. Concurrent speech-language, recreation and aquatic therapies continued.	Participants performed conventional balance activities during baseline and Wii Fit balance-board games during intervention under therapist supervision. Games targeted multidirectional weight shifting, squatting, stepping, single-leg/narrow-base balance, and visually guided postural control. Therapists adjusted difficulty, provided feedback/assistance, and logged game use. Outcomes included dynamic balance (TUG, MFRT), static balance (Wii center-of-pressure ratios), motivation (Pediatric Motivation Scale), and weekly functional ability (PEDI self-care/mobility).	All participants showed basic TUG improvements, though not consistently exceeding baseline rates; MFRT gains varied by direction/arm, with the clearest effect in the longest-treated participant. Static balance was inconclusive (two participants trended toward symmetry; one was unmeasurable due to tremor). Motivation remained high, increasing post-Wii only in one participant. PEDI self-care/mobility improved in all, without a clear dose–response. Findings support feasibility, safety, and engagement, but not superiority over traditional therapy.
Gao et al., 2022 [28]	To evaluate whether receiving inpatient rehabilitation after acute care is associated with better 12-month functional and quality-of-life outcomes than non-inpatient rehabilitation alone in children with severe TBI, and to explore effect modification by discharge GCS	Inpatient rehabilitation (IRF/SNF) vs. non-inpatient rehabilitation (home-based/outpatient). observational comparison of care setting rather than a specific technique	Outcomes assessed at 12 months post-injury; rehabilitation naturalistically received following acute hospitalization (dose/frequency not standardized or reported)	Participants were classified by initial discharge destination: inpatient rehabilitation facility or skilled nursing facility versus direct home discharge with parent-reported rehabilitation use. Outcomes were assessed using the GOS-E Peds (functional/global outcome, 1–8) and the PedsQL (parent- and child-reported health-related quality of life). Confounding was adjusted via propensity-score weighting (inverse probability of treatment) using generalized boosted regression, with discharge GCS tested as an effect modifier.	Among children with GCS ≤ 13 at discharge, inpatient rehabilitation was associated with significantly more favorable global function compared with non-inpatient rehabilitation (adjusted OR = 0.12, 95% CI 0.02–0.95, *p* = 0.045); no such association was observed in children with GCS > 13 (adjusted OR = 1.12, 95% CI 0.51–2.47). Overall, the unadjusted OR was 1.29 (95% CI 0.80–2.09, ns), which reversed to an adjusted OR of 0.75 (95% CI 0.37–1.53, ns) after controlling for confounders. No significant differences emerged between inpatient and non-inpatient groups in parent- or child-reported PedsQL total or domain scores (all *p* > 0.05; differences below minimal clinically important difference thresholds).
Sorek et al., 2023 [29]	To assess cardiac autonomic control-system (CACS) function at rest and during sympathetic and parasympathetic autonomic challenges in children with severe TBI, and to examine changes after 8 weeks of conventional rehabilitation.	Conventional, individualized post-TBI multidisciplinary rehabilitation; the intervention did not specifically target CACS function.	Frequency and intensity were not standardized; treatment was individualized to each child’s physical and functional needs and incorporated multiple rehabilitation approaches and physical activities. CACS testing comprised a 30-min assessment: 5 min of seated rest, a 2-min handgrip challenge, and a 2-min paced-breathing challenge.	Children with severe TBI underwent standard rehabilitation based on developmental, behavioral-neurophysiological, motor-learning, motor-control, and biomechanical principles. Cardiac autonomic function was assessed with a Polar RS800CX during supported sitting (resting), maximal handgrip plus 2-min isometric contraction at 30% (sympathetic reactivity), and paced breathing at 6 breaths/min with biofeedback (parasympathetic/baroreflex reactivity). Primary outcomes were heart rate, SDNN, and RMSSD; secondary outcomes were GOS-E Peds and WeeFIM motor scores.	In 33 children with severe TBI (29 completers) versus 19 controls, baseline resting autonomic function was impaired (higher heart rate; lower SDNN/RMSSD) and remained abnormal after 8 weeks. Challenge tests showed blunted sympathetic (handgrip) and parasympathetic/baroreflex (paced breathing) reactivity at baseline; after rehabilitation, reactivity improved but remained below control levels. Maximal grip strength and respiratory-rate performance also improved. Functionally, GOS-E Peds and WeeFIM scores increased significantly (*p* < 0.001), with most children achieving independent transfers, locomotion, and stair negotiation. Overall, rehabilitation yielded partial recovery of autonomic reactivity during stressors, but not normalization of resting autonomic function.
Stein et al., 2025 [30]	To determine whether a single session of transcranial direct-current stimulation (tDCS) targeting the dorsolateral prefrontal cortex or inferior frontal gyrus, combined with gamified attention training, improves attentional inhibition in children and adolescents with ABI, and to identify neural and anatomical predictors of response.	Bilateral anodal tDCS targeting either the left dorsolateral prefrontal cortex (dlPFC) or right inferior frontal gyrus (IFG), compared with sham stimulation, delivered during computerized/gamified attention training.	Three single-session conditions per participant (dlPFC tDCS, IFG tDCS and sham) administered in counterbalanced order, with 48 h between sessions. Active stimulation lasted 20 min at 1 mA, with 30-s ramp-up and ramp-down periods. Gamified attention training was delivered for 16 min during stimulation.	15 ABI participants (7–18 years) and 15 controls underwent randomized sham-controlled within-subject tDCS: dlPFC (F3/F4) and IFG (F8/F7) using 25-cm^2^ sponges at 0.04 mA/cm^2^; sham was 30 s ramp-up/down. During stimulation, they completed “Wormy Fruit” (Go/No-Go) and “SnappyApp” (CPT). Attention was assessed with Flanker RT and a Stop Signal Task (SSRT). Baseline HD-EEG, optional MRI/diffusion imaging, and electric-field modeling examined connectivity and dose.	In 89 sessions, single-session dlPFC or IFG tDCS showed no group-level benefits over sham for Flanker RT or SSRT in ABI or controls. The ABI group had baseline attentional deficits and altered HD-EEG connectivity. Individual responses varied: slower baseline Flanker RT predicted greater dlPFC-tDCS benefit; lower theta default-mode connectivity predicted faster Flanker after IFG-tDCS; higher gamma default-mode connectivity predicted greater SSRT improvement after dlPFC-tDCS; stronger left-dlPFC electric-field magnitude correlated with faster Flanker RT (*p* = 0.04). Stimulation was well tolerated, with only mild, transient adverse effects and no major safety events.
Furman et al., 2026 [31]	To determine whether greater multidisciplinary therapy intensity during pediatric inpatient rehabilitation is associated with improved functional independence at discharge, shorter length of stay, better motor and cognitive outcomes, and discipline-specific functional gains after acquired brain injury.	Intensive versus lower-intensity multidisciplinary inpatient neurorehabilitation, consisting primarily of occupational therapy, physical therapy and speech-language pathology, with additional neuropsychology, recreational therapy, art counselling and school-based rehabilitation.	The lower-intensity group received a mean of 157.8 direct therapy minutes/day versus 191.0 min/day in the higher-intensity group (difference ≈ 33.2 min/day). Mean length of stay was 20.05 days in the lower-intensity group and 15.16 days in the higher-intensity group, though this difference was not statistically significant.	Participants were matched by age, injury mechanism/characteristics, and admission WeeFIM score. Treatment intensity was calculated as total billable direct-treatment minutes from OT, PT, and SLP divided by length of stay, excluding indirect activities (documentation, meetings, care conferences). Therapy was individualized and goal-directed, targeting functional independence and community reintegration: OT focused on self-care and occupation-based tasks; PT on mobility, motor skills, and physical function; SLP on communication and cognition. WeeFIM outcomes were assessed within 72 h of admission and discharge and expressed as raw scores, efficiency scores, and developmental functional quotients (DFQs).	In 38 matched participants (19 per intensity cohort), both groups improved significantly, but higher versus lower overall intensity did not affect WeeFIM efficiency, discharge scores, or DFQs, nor did total intensity predict length of stay. Greater OT intensity positively predicted total discharge DFQs (+ 0.78 DFQ points per unit; + 21.72% variance explained). PT intensity showed a negative association with discharge DFQs/motor DFQs, likely reflecting treatment escalation for slower recoverers, and explained an additional 44.98% of variance in motor DFQs. Admission DFQs predicted length of stay (29.4% variance in lower-intensity; 51.27% in higher-intensity cohort).
Lippert-grüner & terhaag, 2000 [32]	To describe the use of Multimodal Early Onset Stimulation (MEOS) during early rehabilitation after severe brain injury and to examine physiological and behavioural responses to sensory stimulation in comatose patients.	Multimodal Early Onset Stimulation (MEOS), integrated with early interdisciplinary neurorehabilitation and intensive-care management.	Study period: 2 years. Treatment duration: mean 9.81 days (range 1–30 days) for the 16 patients who fulfilled MEOS criteria. Frequency: two 1-h stimulation blocks per day. Intensity: deliberately controlled, with low noise levels, pauses between stimulation modalities and individualized adjustment according to the patient’s responses and level of consciousness.	The MEOS was delivered in two blocks: Block A (baseline; orofacial, finger/brush, acoustic, visual, olfactory, and gustatory stimulation) and Block B (manual tactile, proprioceptive/kinaesthetic, and thermal stimulation). Acoustic input included conversation, reading, music, and sounds; tactile input involved touch, pressure, massage, cooling, and warming; olfactory and gustatory inputs used familiar odors and flavors; visual input used lights and moving objects; kinaesthetic input involved vestibular and proprioceptive position changes. Physiological responses (heart rate, respiration, blood pressure, galvanic skin response, EEG, EMG) and behavior (Coma Observation Scale) were continuously monitored. Stimulation was individually adapted through a dialogue-based approach rather than a fixed sequence.	Significant stimulus-related changes in heart and respiratory rates were observed even in deeply comatose patients (GCS 3–4), with tactile and acoustic stimulation producing the most consistent physiological effects. In moderate coma (GCS 5–6), these stimuli primarily elicited head and eye movements, while behavioral assessment became more informative as consciousness improved. The study did not provide a controlled quantitative estimate of treatment-related neurological recovery, and the authors cautioned that physiological responsiveness should not be interpreted as evidence of a favorable prognosis. At 2-year follow-up (*n* = 14), outcomes were: one patient remained in a VS (GOS 2); two had severe deficits and remained care-dependent (GOS 3); six had severe functional limitations but were independent in daily activities (GOS 4); and three achieved good recovery (GOS 5), including two who returned to their previous employment.
Zheng et al., 2025 [33]	To evaluate the efficacy and safety of 5 Hz rTMS over the left DLPFC for consciousness recovery in children with DOC after TBI.	5 Hz rTMS plus conventional rehabilitation versus conventional rehabilitation alone.	Once daily for 3 consecutive weeks; 1000 pulses/session; 20 min/session at 80% RMT.	Randomized controlled trial. The experimental group received rTMS plus conventional rehabilitation, including ROM exercises, sensory stimulation, and acupuncture.	The experimental group showed greater improvement in CRS-R, GCS, NSE, and level of consciousness than the control group. No adverse events were reported.
Smith et al., 2025 [34]	To examine whether participation in intensive, time-limited Parent–Child Interaction Therapy (PCIT) is associated with reductions in caregiver-reported sleep problems among preschool children who sustained a TBI, and whether changes are maintained at a 2-month follow-up.	Intensive, time-limited Parent–Child Interaction Therapy (PCIT), delivered in English or Spanish and provided in clinic or at home according to family needs.	10 sessions of 60–90 min delivered twice weekly over a mean of ~6 weeks. Intensity was increased via the twice-weekly, time-limited format, without requiring caregivers to meet standard mastery criteria before progression.	PCIT comprised two phases: CDI (PRIDE skills; reduced Questions/Commands/Negative Talk) and PDI (effective commands, limit-setting, positive discipline). Each phase included one teaching session plus four live coaching sessions during caregiver–child play. Two families received home-based treatment with flexible scheduling. The intervention did not target sleep or include bedtime coaching. Therapists were doctoral-level, supervised by a licensed PCIT trainer; sessions were recorded, and fidelity was 99%.	Caregiver-reported CBCL Sleep Problems scores fell from 9.00 at baseline to 3.56 post-intervention (mean reduction 6.50; 95% CI 3.42–7.45; *p* < 0.001), indicating a large effect, with gains maintained at 2-month follow-up (post M = 4.08 vs. follow-up M = 4.17; *p* = 0.926). Four of six children (66.7%) showed reliable improvement post-treatment; four of five (80%) at follow-up. Clinically significant change (T ≥ 60 to <60) occurred in 4/6 post-treatment (66.7%) and 3/5 at follow-up (60%); overall, 5/6 (83.3%) showed reliable or clinically significant improvement at one or more assessments. Caregiver parenting improved: CDI “Do” skills rose from 3.67 to 14.17 post-treatment (follow-up 9.83), while “Don’t” skills fell from 39.17 to 17.33 (follow-up 27.67).

Legend: activities of daily living (ADL); acquired brain injury (ABI); Besta neurological scale (Besta Scale); constraint-induced movement therapy (CIMT); Coma Recovery Scale-Revised (CRS-R); dorsolateral prefrontal cortex (DLPFC); disorders of consciousness (DOC); electropalatography (EPG); Functional Independence Measure (FIM); Glasgow Coma Scale (GCS); Glasgow Outcome Scale (GOS); Gross Motor Function Measure (GMFM); heart rate variability (HRV); inferior frontal gyrus (IFG); Manual Ability Classification System (MACS); multimodal early onset stimulation (MEOS); neuron-specific enolase (NSE); occupational therapy (OT); parent–child interaction therapy (PCIT); physical therapy (PT); quality of life (QoL); resting motor threshold (RMT); repetitive transcranial magnetic stimulation (rTMS); speech-language pathology (SLP); traumatic brain injury (TBI); transcranial direct current stimulation (tDCS); vegetative state (VS).

## Data Availability

Data reported in this study are available from the corresponding author on reasonable request.

## Supplementary Materials

The following supporting information can be downloaded at: https://www.mdpi.com/article/10.3390/jcm15197645/s1: File S1: PRISMA-ScR-Checklist [48].

## Abbreviations

The following abbreviations are used in this manuscript:

TBI	Traumatic brain injury
Doc	Disorders of consciousness
VS	Vegetative state
USW	Unresponsive wakefulness syndrome
MCS	Minimally conscious state
ABI	Acquired brain injury
CIMIT	Constraint-induced movement therapy
PCIT	Parent–child interaction therapy
rTMS	Repetitive transcranical magnetic stimulation
MACS	Manual Ability Classification System
FIM	Functional independence measure
GOS	Glasgow Outcome Scale
